# Small bowel hemorrhage from check point inhibitor enteritis: a case report

**DOI:** 10.1186/s12876-021-01915-1

**Published:** 2021-09-07

**Authors:** Kellie Young, Emery Lin, Emerson Chen, Brian Brinkerhoff, Gregory Scott, Jessica Yu

**Affiliations:** 1 Division of Gastroenterology and Hepatology, Oregon Health and Science University, Portland, OR USA; 2 Department of Gastroenterology and Hepatology, The Portland VA Medical Center, Portland, OR USA; 3 Division of Hematology and Medical Oncology, Oregon Health and Science University, Knight Cancer Institute, Portland, OR USA; 4 Department of Pathology, Oregon Health and Science University, Portland, OR USA

**Keywords:** Immune checkpoint enteritis, Gastrointestinal hemorrhage, Methylprednisolone, Infliximab, Case report

## Abstract

**Background:**

There is rising utilization of immune checkpoint inhibitors (ICI) for a growing number of metastatic malignancies. While gastrointestinal side effects of ICI are common, isolated ICI-induced enteritis leading to small bowel hemorrhage is rare.

**Case presentation:**

A 71-year-old man with a previously resected right colon adenocarcinoma on atezolizumab and recently treated *Clostridioides difficile* presented with acute on chronic abdominal pain and non-bloody diarrhea. A CT scan revealed enteritis of the duodenum and jejunum without colitis. Initial endoscopic work-up revealed many clean-based non-bleeding duodenal ulcers to the third portion of the duodenum and normal rectosigmoid mucosa. The patient initially improved on steroids but was readmitted on day after discharge with hematochezia and hemorrhagic shock. Repeat CT showed improvement in enteritis; however, repeat push enteroscopy revealed multiple duodenal and jejunal ulcers, two with visible vessels requiring endoscopic intervention. He continued to have significant hemorrhage requiring transfusions despite IV methylprednisolone. Conventional angiogram revealed multiple sites of active extravasation, and he underwent small bowel resection and subsequent IR embolization due to persistent bleeding. He was then started on infliximab 10 mg/kg with resolution of his small bowel hemorrhage and diarrhea.

**Conclusions:**

Severe isolated ICI-enteritis is rare and can lead to clinically significant gastrointestinal hemorrhage. Patients with severe ICI-enteritis on endoscopy should be carefully monitored for steroid refractory disease for consideration of step-up therapy such as infliximab.

## Background

Immune checkpoint inhibitors (ICI) have increasing roles in the treatment of metastatic malignancies such as melanoma, Hodgkin’s disease, renal cell carcinoma, and hepatocellular carcinoma [1, 2]. These medications target cytotoxic T-lymphocyte-associated antigen 4 (CTLA-4) (e.g. ipilimumab), programmed cell death 1 (PD1) (e.g. nivolumab, pembrolizumab) and anti-programmed death-ligand 1 (PD-L1) (atezolizumab, avelumab) [1, 2]. Common gastrointestinal side effects of ICI include diarrhea and colitis; however, gastrointestinal bleeding is less well described. To our knowledge, there have been no previously reported cases of severe ICI-induced enteritis leading to significant gastrointestinal hemorrhage.

## Case presentation

**Fig. 1:**
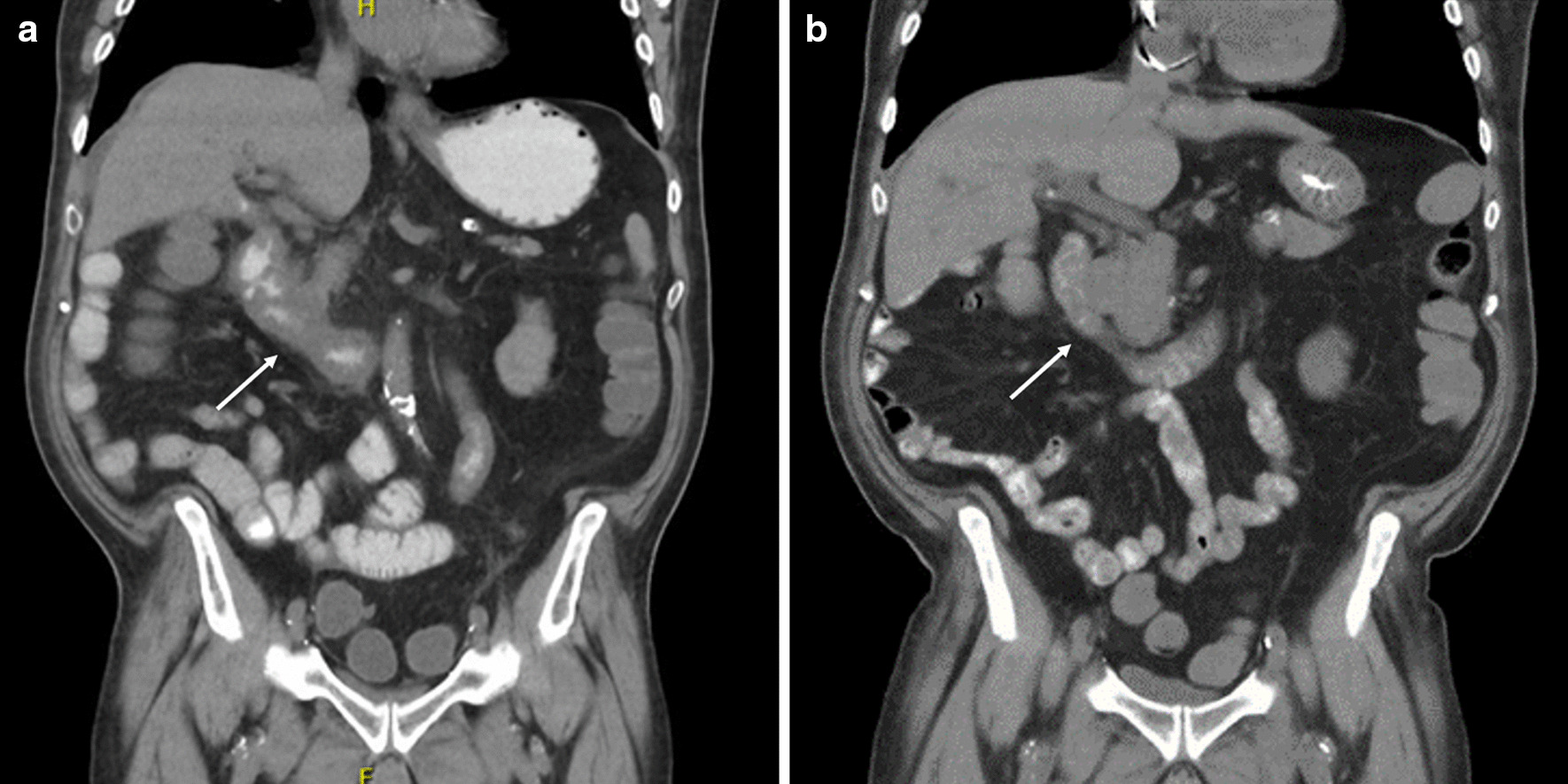
**a** CT abdomen/pelvis on initial presentation with enteritis and lack of colitis. **b** CT abdomen/pelvis on second presentation with hemorrhagic shock, showing improved enteritis

A 71-year-old man with a history of a resected right colon adenocarcinoma, Stage IIIB (pT3, pN1b, cM0) on atezolizumab, was admitted to the hospital with acute on chronic abdominal pain and non-bloody diarrhea. The patient was on oral vancomycin due to a positive *Clostridioides difficile* test 6 days prior to admission which had not improved his diarrhea and repeat stool testing on admission was negative. A CT scan revealed a normal colon and thickening of multiple loops of duodenum and proximal jejunum with mesenteric edema, consistent with enteritis (Fig. 1a). An upper endoscopy showed many clean-based non-bleeding duodenal ulcers to the third portion of the duodenum which were biopsied. Biopsies showed gastric foveolar metaplasia and no evidence of CMV infection. Flexible sigmoidoscopy was endoscopically and histologically normal. Although the duodenal biopsies were non-specific, his bowel movement frequency (5–6 a day) was concerning for grade 2 ICI-induced enteritis. IV methylprednisolone was initiated with improvement in his symptoms. He was thus transitioned to prednisone and discharged.

**Fig. 2:**
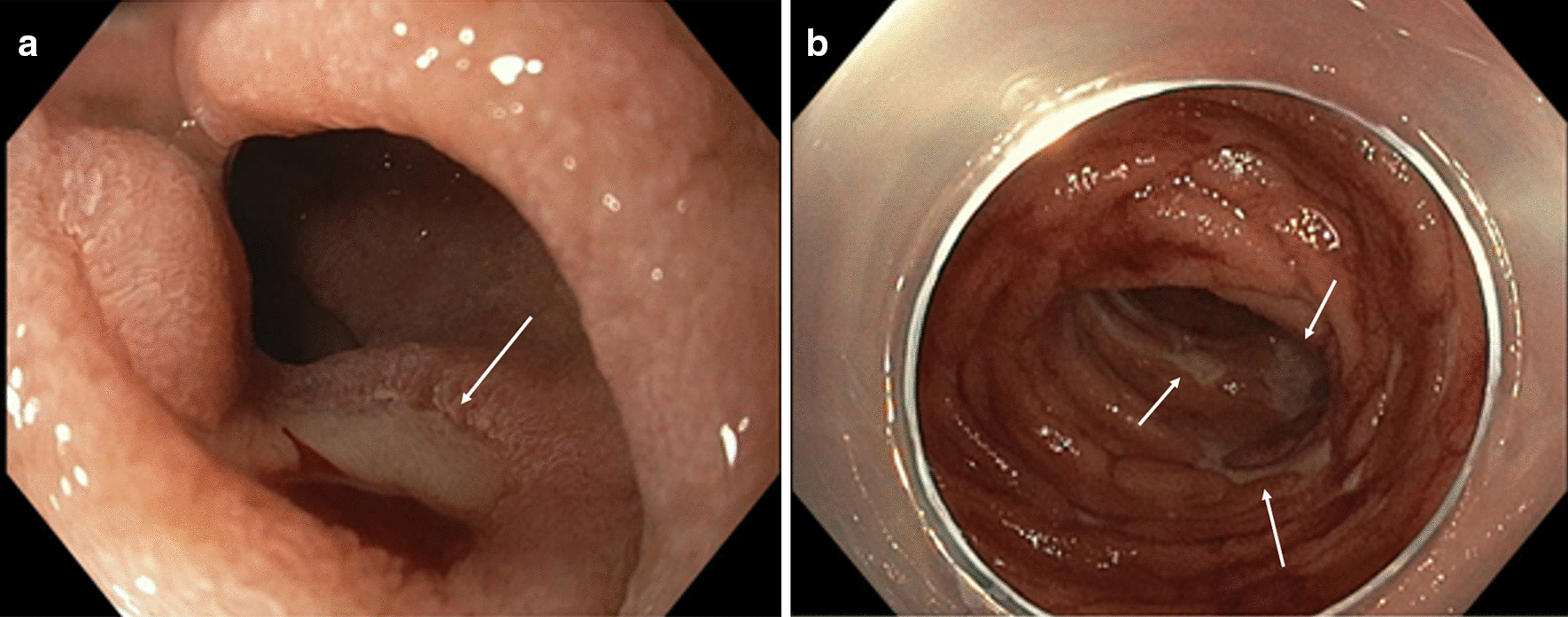
**a** Ulcer seen on push enteroscopy with active oozing. **b** Multiple ulcers with fibrinous base seen throughout small intestines on push enteroscopy

**Fig. 3:**
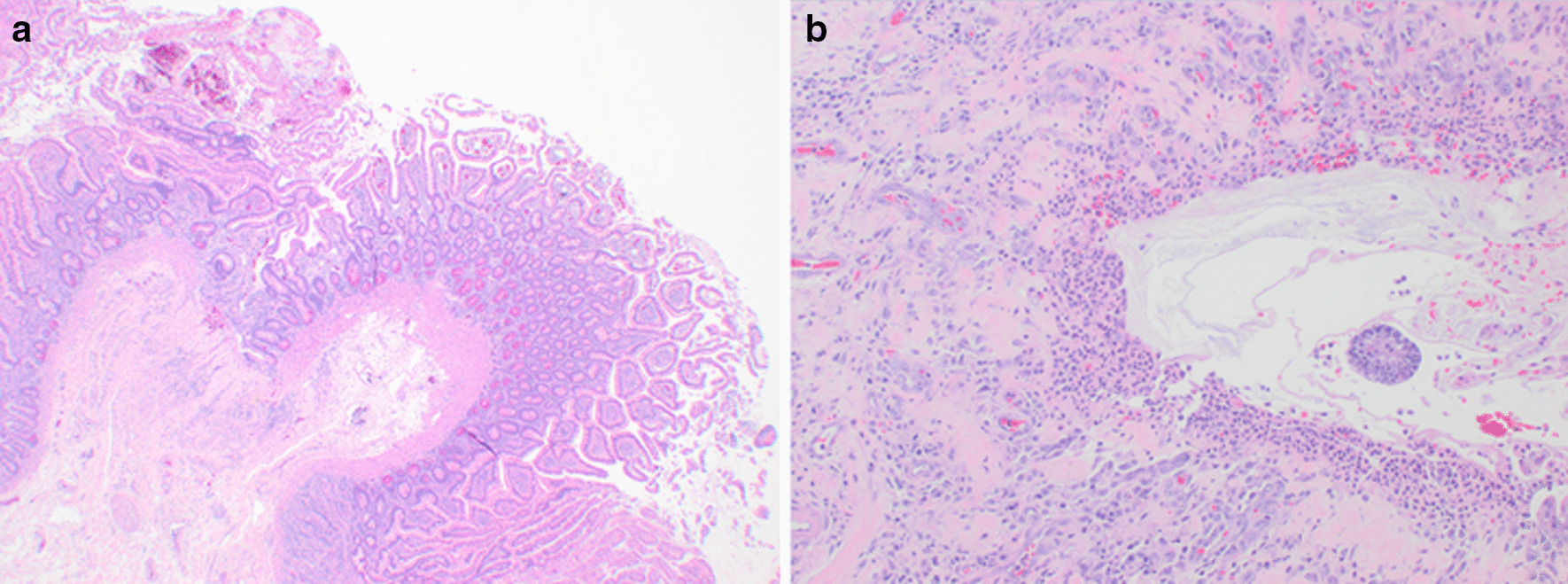
**a** Microscopic view of jejunal resection showing small bowel mucosa with subtle ischemic change (low-power view). **b** Microscopic view of mucosal erosion with underlying granulation tissue, compatible with clinical picture of medication-induced enteritis (high-power view)

He presented one day after discharge with hematochezia and hemorrhagic shock. CT showed overall improvement in duodenal and proximal jejunal thickening (Fig. 1b). A push enteroscopy demonstrated multiple duodenal and jejunal ulcers (Fig. 2), two with visible vessels which were treated with epinephrine, hemoclips and bipolar cautery. Despite endoscopic therapy and re-initiation of IV methylprednisolone, he continued to have clinically significant bleeding. IR angiogram revealed active extravasation at multiple sites in the proximal jejunum for which he underwent a small bowel resection with removal of 40 cm of ulcerated jejunum. Pathology showed scattered mucosal erosions with active enteritis and ischemic-type changes, with multifocal areas of denudation and granulation tissue. There was no evidence of vasculitis, fungal elements, or viral staining for CMV or adenovirus (Fig. 3). He continued to have melena with transfusion requirements after his small bowel resection, requiring inferior pancreaticoduodenal artery embolization. Given his progression to Grade 4 ICI-induced enteritis, he was loaded with infliximab 10 mg/kg with resolution of his GI bleeding 2 days after his infusion. He underwent one additional outpatient dose of infliximab and has had no further bleeding episodes and reported resolution of his diarrhea on his most recent clinic follow up.

## Discussion and conclusions

We describe a case of severe isolated ICI-induced enteritis that progressed rapidly despite high dose steroids, leading to hemorrhagic shock.

The most common ICI-induced gastrointestinal adverse events are colitis (8.4–11.3% with anti-CTLA-4, 0.3–3.4% with anti-PD1, and up to 14% in combination) and diarrhea (35–40% with anti-CTLA-4, 11–17% with anti-PD1, and 32% with combination therapy) [1, 3, 4]. Initial management of ICI-induced diarrhea depends on the grade at presentation. For Grade 1 (increase in < 4 stools a day over baseline), anti-diarrheal medications such as loperamide with a low fiber diet can be started with ICI therapy maintained. For Grade 2 (increase in 4–6 stools a day over baseline), ICI therapy should be held with initiation of oral steroids. For Grade 3 (increase of ≥ 7 stools a day over baseline) or Grade 4 (life threatening symptoms with urgent intervention required), IV steroids should be initiated in addition to holding ICI therapy [1]. Recurrence of ICI-induced gastrointestinal adverse events occurs in 23–32% of cases, if patients are re-treated with ICI therapy. There is also a higher risk associated with immunosuppressive therapy required during the first episode, anti-PD1 therapy as the first line agent, and anti-CTLA-4 utilized as the second-line agent [1].

ICI-induced gastrointestinal hemorrhage and management is less well defined in the literature. A retrospective study that focused on patients with gastrointestinal side effects from PD-1 blockade reported 10% with hematochezia. However, all these patients’ hematochezia was a result from PD-1 associated acute colitis. Of the 20 patients included in the study, only one had terminal ileitis and none had duodenal involvement [5].

While ICI-associated enteritis has been described, it uncommonly occurs in isolation from colitis or with severe ulceration leading to clinically significant GI bleeding. When isolated enteritis is present, patients’ main presentation is diarrhea. Of the existing published case reports of isolated enteritis, ICI regimens utilized were ipilimumab or nivolumab in mono or combination therapy [6–8]. To our knowledge, this is the first reported case of severe GI hemorrhage from ICI-induced enteritis. As our patient’s bleeding was refractory to corticosteroid therapy, prompt initiation of infliximab, an anti-tumor necrosis factor (anti-TNF) was indicated. Risk factors for steroid-refractory ICI-induced colitis have been described in patients with pancolitis and with severe endoscopic exams (Mayo 2–3), and should be considered in patients whose GI symptoms fail to respond after 3–5 days of high dose steroids [1]. Thus, similar considerations for early infliximab initiation should be given to patients who demonstrate significant endoscopic small bowel involvement, who may be at higher risk of steroid refractory disease and clinical deterioration.

## Data Availability

Not applicable.

## Abbreviations

Anti-TNF: Anti-tumor necrosis factor
ICI: Immune checkpoint inhibitor
CT: Computed tomography
CTLA-4: Cytotoxic T-lymphocyte-associated antigen 4 (CTLA-4)
PD1: Programmed cell death 1 (PD1)
PD-L1: Anti-programmed death-ligand 1
